# Supplementary material for: Role of ivermectin in the prevention of SARS-CoV-2 infection among healthcare workers in India: A matched case-control study
Source: PLoS One. 2021 Feb 16;16(2):e0247163. doi: 10.1371/journal.pone.0247163 (PMC7886121; doi:10.1371/journal.pone.0247163)
Supplement: S1 Appendix — (PDF) [file pone.0247163.s001.pdf]

Study Questionnaire

1. SRF ID :
2. NAME :
3. AGE :
4. GENDER: Male ( ) Female ( )
5. CASE: ( ) CONTROL ( )
6. Designation :
   A. Faculty ( )
   B. Resident ( )
   C. Intern ( )
   D. UG Student ( )
   E. Nursing Officer ( )
   F. Other Nursing Staff ( )
   G. AYUSH ( )
   H. Supportive Staff ( )
   I. Other ( )

7. Household- Currently Staying With?

   A. Alone/Hostel ( )
   B. Nuclear Family ( )
   C. Extended/Joint Family ( )
   D. Friends/Roommate ( )

8.COVID-19 RTPCR Status: have you ever tested positive for COVID-19? Yes ( ) no ( )

10. Did you have COVID related duty? YES ( ) NO ( )

11.Duration of duty (no. Of days):………………

12.Number of times posted (no. Of rotations):……………..

13.Did you have any symptoms? YES ( ) NO ( )

14. If yes, what were your symptoms?

   A. Fever ( )
   B. Cold ( )
   C. Cough ( )
   D. Sore Throat ( )
   E. Myalgia ( )
   F. Diarrhoea ( )
   G. Headache ( )
   H. Breathlessness ( )
   I. Chest Pain ( )
   J. Loss Of Taste/Smell ( )
   K. Weakness ( )
   L. Skin Rash ( )
   M. Conjunctivitis ( )
   N. Discolouration Of Toes/Fingers ( )
   O. Other ( ) If Others Specify…………………

15. Any history of contact with positive case? YES ( ) NO ( )

16.if yes, what type of contact?

A. Home (Family Members/Roommate)   ( )
B. Social (Friends/Public Place etc.) ( )
C. Duty (Workplace- Co-worker/Patient etc.) ( )

17.Opted for which type of isolation?

A. Home Isolation ( )
B. Institutional Isolation ( )
C. Hospital Admission ( )

18. If hospitalised, how many days admitted?:……………….

19. Did any of your contacts test positive?   YES ( )   NO ( )

20. How many close contacts did you have after coming positive?................

21. How many of your contacts also tested positive, if any?...................

HISTORY OF ANY PROPHYLAXIS

22. Did you take any prophylaxis for covid-19? YES ( )   NO ( )

23. If yes, what did you take for prophylaxis?

A. Ivermectin ( )
B. Hydroxychloroquine ( )
C. Vitamin C ( )
D. Ayurvedic/Homeopathic Therapy ( )
E. Other ( )   If Others Specify…………………

24. If Ivermectin, then dose?

A. 12 MG ( )
B. 15 MG ( )
C. 18 MG ( )
D. 24 MG ( )

25. If Ivermectin, no. Of days ivermectin taken?................

26. If Hydroxychloroquine, how much dosage? ………………

27. If HCQ, no. Of days HCQ taken? ………………..

28. If Vitamin C taken, then dose?

A. 500 MG OD ( )
B. 500 MG BD ( )

29. If vitamin-C taken, no. of days/duration vitamin-C taken? ………………..

30. If any other prophylaxis, please specify what?.......................

31. If yes, please specify how much dose and type of preparation?..

32. For how many days have you been taking the prophylaxis?.....................

PHYSICAL ACTIVITY

33. Physical activity: YES ( ) NO ( )

34. Type of physical activity?

A. Walking ( )
B. Jogging ( )
C. Yoga ( )
D. Gym ( )
E. Sports ( )
F. Other ( ) If Others Specify…………………

35. If yes, no. of days per week?................

36. If yes, no. of minutes/day?................

Study Questionnaire Odia (local language)

1. ଏସ ଆର ଏଫ:
2. ନାମ:
3. ବୟସ:
4. ଲିଙ୍ଗ: ପୁରୁଷ ( ) ମହିଳା ( )
5. କେସ ( ) କଂଟ୍ରୋଲ ( )
6. ପଦବୀ:

   A. ଶିକ୍ଷକ ( )
   B. ରେସିଡେଣ୍ଟ ( )
   C. ଇଂଟର୍ନ ( )
   D. ୟୁଜି ଛାତ୍ର ( )
   E. ନର୍ସିଙ୍ଗ ଅଫିସର ( )
   F. ଅନ୍ୟ ନର୍ସିଂ କର୍ମଚାରୀ ( )
   G. ଆୟୁଷ ( )
   H. ସହାୟକ କର୍ମଚାରୀ ( )
   I. ଅନ୍ୟମାନେ ( )

7. ଘର-ସମ୍ପ୍ରତି କାହା ସହିତ ରୁହନ୍ତି?

   A. ଏକାକୀ / ହଷ୍ଟେଲ ( )
   B. ଏକକ ପରିବାର ( )
   C. ବିସ୍ତାରିତ / ମିଳିତ ପରିବାର ( )
   D. ବନ୍ଧୁ / ରୁମମେଟ ( )

8. କୋଭିଡ-୧୯ ଆର.ଟି.ପି.ସି.ଆର. ସ୍ଥିତି: ଆପଣ କେବେ କୋଭିଡ-୧୯ ପାଇଁ ପଜିଟିଭ୍ ଚିହ୍ନଟ ହେଇଛନ୍ତି କି? ହଁ ( ) ନା ( )

10. ଆପଣଙ୍କର କୋଭିଡ-୧୯ ସମ୍ବନ୍ଧୀୟ ଦାୟିତ୍ୱ ଥିଲା କି?? ହଁ ( ) ନା ( )

11. ଡ୍ୟୁଟିର ଅବଧି (ଦିନ ସଂଖ୍ୟା):………………

12. ପୋଷ୍ଟିଂର ସଂଖ୍ୟା (ଶିଫ୍ଟ ସଂଖ୍ୟା): …………… ..

13. ଆପଣଙ୍କର କୌଣସି ଲକ୍ଷଣ ଅଛି କି? ହଁ ( ) ନା ( )

14. ଯଦି ହଁ, ଆପଣଙ୍କର ଲକ୍ଷଣଗୁଡିକ କ'ଣ ଥିଲା?

   A. ଜ୍ୱର ( )
   B. ଥଣ୍ଡା ( )
   C. କାଶ ( )
   D. ଗଳା ଖରାପ'( )
   E. ମାଂସପେଶୀ ଯନ୍ତ୍ରଣା ( )
   F. ଡାଇରିଆ ( )
   G. ମୁଣ୍ଡବିନ୍ଧା ( )
   H. ନିଶ୍ୱାସ ନେବାରେ ଅସୁବିଧା ( )
   I. ଛାତି ଯନ୍ତ୍ରଣା ( )
   J. ଗନ୍ଧ କିମ୍ବା ସ୍ୱାଦରେ ଅସୁବିଧା ( )

K. ଦୁର୍ବଳତା ( )
L. ଚର୍ମ ଦାଗ ( )
M. କଂଜୁଙ୍କଟିଭିଟିସ ( )
N. ଆଙ୍ଗୁଠିର ରଙ୍ଗୀନତା ( )
O. ଅନ୍ୟାନ୍ୟ ( ),ଯଦି ଅନ୍ୟାନ୍ୟ ତାହେଲେ ନିର୍ଦ୍ଦିଷ୍ଟ କରନ୍ତୁ ....

15. କୋଭିଡ-୧୯ ପଜିଟିଭ୍ ମାମଲା ସହିତ ଯୋଗାଯୋଗର କୌଣସି ଇତିହାସ? ହଁ ( ) ନା ( )

16. ଯଦି ହଁ, କେଉଁ ପ୍ରକାର ସମ୍ପର୍କ?
A. ଗୃହ (ପରିବାର ସଦସ୍ୟ / ରୁମମେଟ) ( )
B. ସାମାଜିକ (ବନ୍ଧୁ / ସର୍ବସାଧାରଣ ସ୍ଥାନ ଇତ୍ୟାଦି) ( )
C. କର୍ତ୍ତବ୍ୟ (କର୍ମକ୍ଷେତ୍ର- ସହକର୍ମୀ / ରୋଗୀ ଇତ୍ୟାଦି) ( )

17. କେଉଁ ପ୍ରକାରର ଆଇସୋଲସନ ପାଇଁ ଚୟନ କରାଯାଇଛି?
A. ହୋମ ଆଇସୋଲସନ ( )
B. ଅନୁଷ୍ଠାନିକ ଆଇସୋଲସନ ( )
C. ହସ୍ପିଟାଲ୍ ଆଡମିଶନ ( )

18. ଯଦି ତୁମେ ହସ୍ପିଟାଲରେ ଭର୍ତ୍ତି ହୋଇଛ, ତୁମକୁ କେତେ ଦିନ ଆଡମିଶନ କରାଯାଇଛି?:..................

19. ଆପଣଙ୍କର କୌଣସି ସମ୍ପର୍କ କୋଭିଡ-୧୯ ପଜିଟିଭ୍ ଚିହ୍ନଟ ହେଇଛନ୍ତି କି? ହଁ ( ) ନା ( )

20. ସକରାତ୍ମକ ଆସିବା ପରେ ତୁମର କେତେ ଜଣଙ୍କ ସଂଗେ ଘନିଷ୍ଠ ସମ୍ପର୍କ ଥିଲା??................

21. ଆପଣଙ୍କର କେତେ ଜଣ ସମ୍ପର୍କ କୋଭିଡ-୧୯ ପଜିଟିଭ୍ ଚିହ୍ନଟ ହେଇଛନ୍ତି?.................

HISTORY OF ANY PROPHYLAXIS

22. କୋଭିଡ-୧୯ ପାଇଁ ଆପଣ କୌଣସି ପ୍ରୋଫାଇଲକ୍ସିସ୍ ନେଇଛନ୍ତି କି? ହଁ ( ) ନା ( )

23. ଯଦି ହଁ, ପ୍ରୋଫାଇଲକ୍ସିସ୍ ପାଇଁ ଆପଣ କ'ଣ ନେଇଥିଲେ?
A. ଆଇଭେରମେକ୍ଟିନ ( )
B. ହାଇଡ୍ରୋକ୍ସିକ୍ଲୋରୋକାଇନ୍ ( )
C. ଭିଟାମିନ୍ ସି ( )
D. ଆୟୁର୍ବେଦିକ / ହୋମିଓପାଥିକ୍ ଥେରାପି ( )
E. ଅନ୍ୟାନ୍ୟ ( ),ଯଦି ଅନ୍ୟାନ୍ୟ ତାହେଲେ ନିର୍ଦ୍ଦିଷ୍ଟ କରନ୍ତୁ ....

24. ଯଦି ଆଇଭେରମେକ୍ଟିନ, କେତେ ମାତ୍ରା ?
A. 12 MG ( )
B. 15 MG ( )
C. 18 MG ( )
D. 24 MG ( )

25. ଯଦି ଆଇଭେରମେକ୍ଟିନ, କେତେ ଦିନ ଆଇଭେରମେକ୍ଟିନ ନିଆଯାଇଛି?...............

26. ଯଦି ହାଇଡ୍ରୋକ୍ସିକ୍ଲୋରୋକାଇନ୍, କେତେ ମାତ୍ରା ....

27. ଯଦି ହାଇଡ୍ରୋକ୍ସିକ୍ଲୋରୋକାଇନ୍, କେତେ ଦିନ ନିଆଯାଇଛି? ....

28. ଯଦି ଭିଟାମିନ୍ ସି ନିଆଯାଇଛି, ତେବେ ଏହାର ମାତ୍ରା?
A. 500 MG OD ( )
B. 500 MG BD ( )

29. ଯଦି ଭିଟାମିନ୍ ସି, କେତେ ଦିନ ଭିଟାମିନ୍ ସି ନିଆଯାଇଛି? ....

30. ଯଦି ଅନ୍ୟ କୌଣସି ପ୍ରୋଫାଇଲକ୍ସିସ୍ ନେଉଛନ୍ତି, ଦୟାକରି କ'ଣ ନିର୍ଦ୍ଦିଷ୍ଟ କରନ୍ତୁ? ....

31. ଯଦି ହଁ, ଦୟାକରି ନିର୍ଦ୍ଦିଷ୍ଟ କରନ୍ତୁ କେତେ ପରିମାଣର ପ୍ରସ୍ତୁତି ଏବଂ ପ୍ରକାର ନେଉଛନ୍ତି? ....

32. ଆପଣ କେତେ ଦିନ ଧରି ଏହି ପ୍ରୋଫାଇଲକ୍ସିସ୍ ନେଉଛନ୍ତି? ....

PHYSICAL ACTIVITY

33. ଶାରୀରିକ କାର୍ଯ୍ୟକଳାପ: ହଁ ( ) ନା ( )

34. ଶାରୀରିକ କାର୍ଯ୍ୟକଳାପର ପ୍ରକାର:

A. ଚାଲିବା ( )
B. ଜଗିଂ ( )
C. ଯୋଗ ( )
D. ଜିମ୍ ( )
E. କ୍ରୀଡା ( )
F. ଅନ୍ୟ ( ) ଯଦି ଅନ୍ୟାନ୍ୟ ତାହେଲେ ନିର୍ଦ୍ଦିଷ୍ଟ କରନ୍ତୁ ....

35. ଯଦି ହଁ, ସପ୍ତାହରେ ଦିନ ସଂଖ୍ୟା? ....

36. ଯଦି ହଁ, ପ୍ରତିଦିନ ମିନିଟ୍ ସଂଖ୍ୟା? ....
