# Supplementary material for: Role of ivermectin in the prevention of SARS-CoV-2 infection among healthcare workers in India: A matched case-control study
Source: PLoS One. 2021 Feb 16;16(2):e0247163. doi: 10.1371/journal.pone.0247163 (PMC7886121; doi:10.1371/journal.pone.0247163)
Supplement: S1 Protocol — (DOC) [file pone.0247163.s002.doc]

**Protocol**

**Title: Role of ivermectin in the prevention of COVID19 disease among health care workers in India: A case-control Study**

**Primary Investigator**

Dr. Priyamadhaba Behera

Assistant Professor

Department of Community Medicine and Family Medicine

All India Institute of Medical Sciences, Bhubaneswar

Email: [cmfm_priyamadhaba@aiimsbhubaneswar.edu.in](mailto:cmfm_priyamadhaba@aiimsbhubaneswar.edu.in)

Mobile: +91 9910830997

1. **Introduction:**

An outbreak of novel coronavirus disease (COVID-19) was reported in the Wuhan province of China in December 2019, which has become a pandemic with infection being reported from 213 countries across the globe as of 10 September 2020. This is still an evolving pandemic, and at the time of writing this proposal on 10 September 2020, there were 27.9 million reported cases with 0.9 million deaths [1].

Healthcare workers are on the front lines of the global effort to care for patients with COVID-19 while putting themselves at risk for infection. Thousands have already died from dozens of countries, professions, and specialties. As of 1 July, this list includes more than 1800 health care workers from 64 countries who have lost their lives. The youngest is 20 years old, and the eldest is 99 years old [2].

Ivermectin, which is an FDA-approved broad-spectrum anti-parasitic agent, also has antiviral activity. It has antiviral activity against several animal and human viruses, including both RNA and DNA viruses. The antiviral potential of ivermectin against various viruses is mediated via the targeting of the following: importin α/β-mediated nuclear transport of HIV-1 integrase and NS5 polymerase; NS3 helicase; nuclear import of UL42; and nuclear localization signal mediated nuclear import of Cap [3-7]. In vitro study has shown its activity against SARS-CoV-2 [8]. It was proposed that it involves the binding of ivermectin to the Impα/β1 heterodimer, leading to its destabilization and prevention of Impα/ β1binding to the viral proteins. This prevents viral proteins from entering the nucleus, thereby reducing the inhibition of antiviral responses and leading to an efficient antiviral response [8].

The safety of ivermectin has been proved beyond doubt in large large-scale disease eradication programs. In the past 21 years, more than 1 billion doses of ivermectin tablets have been distributed for both onchocerciasis and filariasis, at doses of 100-200 µg/kg. [9-10]. However, whether ivermectin can be used as prophylaxis for COVID-19 disease? If yes, what will be the possible dose in a real-life context? The available literature is limited in the above context [11]. Therefore, our study aimed to study the role of oral ivermectin for the prevention of COVID-19 disease and explore the probable dose, which can be useful in this context.

**2. Research Question**

This study is designed to answer the following questions –

2.1 Whether oral ivermectin can be used for the prevention of COVID-19 disease?

- 1. If so, what is the probable dose of ivermectin which will benefit maximum in the Indian context?

**3. Objectives:**

3.1 Primary Objective: To find the association between oral ivermectin prophylaxis and COVID-19 infection among healthcare workers.

3.2 Secondary Objective: To explore the possible prophylaxis dose of ivermectin for the prevention of COVID-19 disease.

**4. Materials and methods:**

**4.1 Study area:** AIIMS Bhubaneswar

**4.2 Study population:** Health care workers of AIIMS, Bhubaneswar

**4.3 Study design:** Matched Case-control study

**4.4 Study duration:** 2 months (September 2020-October 2020)

**4.5 Eligibility criteria:**

*Inclusion criteria:*

- The persons should have worked (permanent/contractual) in the AIIMS, Bhubaneswar
- Should provide written informed consent (face to face interview)/Telephonic consent (for telephonic interview) for participation in the study.

*Exclusion criteria:*

Persons who will not provide consent for participation of the study.

**4.6. Definition of the case, control and exposure:**

**Case:** Healthcare worker who was diagnosed positive for COVID-19 by RTPCR in last one month.

**Control:** Healthcare workers who are free from COVID-19 as declared by RTPCR in the last one month.

**Exposure:** Exposure is oral ivermectin received in any dose.

**4.7. Sample size:**

Considering, 80% power, 5% alpha, 1:1 matching of cases to controls, minimum discordant pairs to be detected was set to 54, with an expected odds ratio of 0.5 and one side hypothesis, the sample size was estimated to be 186 pairs, i.e., 372 individuals. The sample size was calculated using nMaster 2.0 software [12].


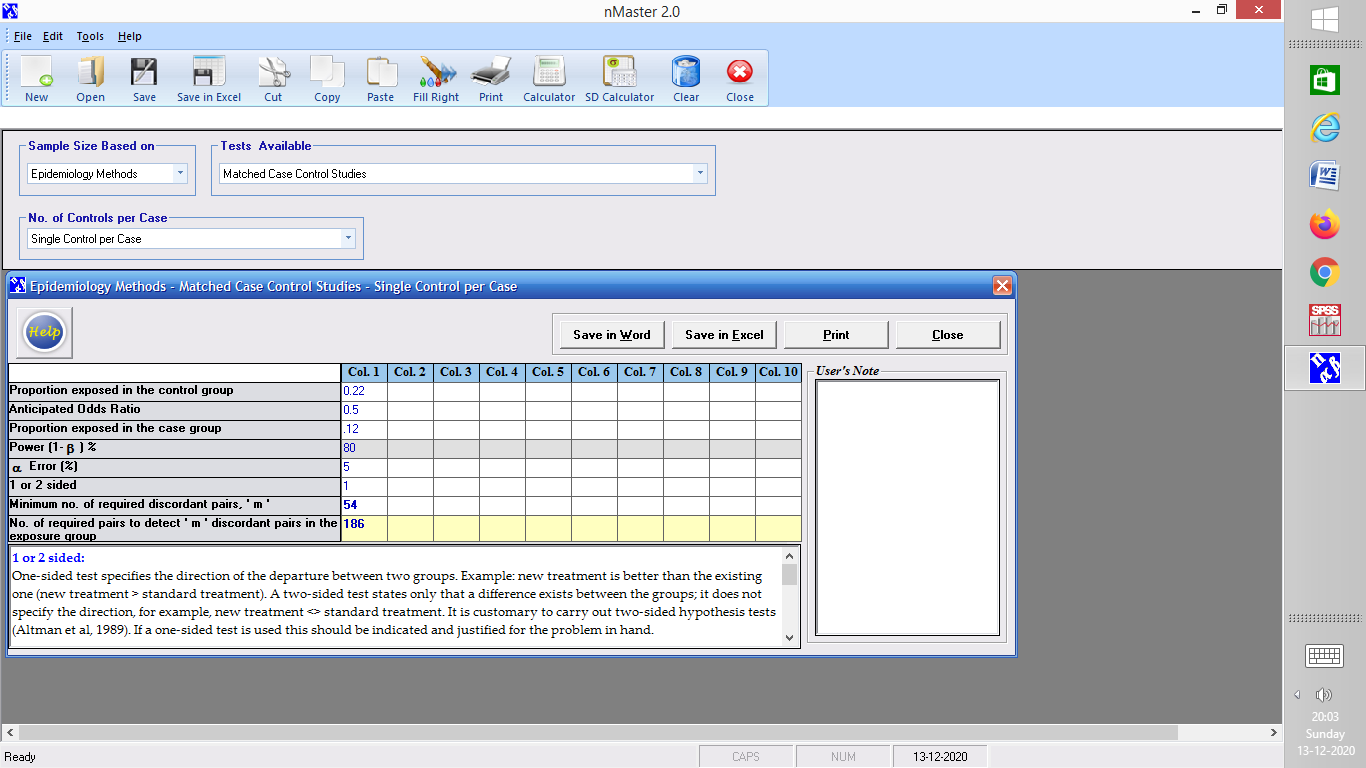


**4.8 Study Procedure:** Healthcare worker who was diagnosed positive for COVID19 by RTPCR in the last one month will be identified from the record review. One hundred eighty-six COVID-19 positive cases will be identified. One hundred eighty-six controls (RT PCR negative for COVID19) will be chosen for each case after matching with the profession, age, sex and date of diagnosis of COVID-19. Hence, there will 186 cases and 186 controls that will be telephonically interviewed. All the participants (372) will be asked about the history of consumption of any prophylaxis i.e ivermectin, HCQ, vitamin-C or any other.

**4.9. Ethical consideration:** Ethical approval will be taken from the Institute Ethics Committee of All India Institute of Medical Sciences, Bhubaneswar. Informed consent (Telephonic) will be taken from the participants after explaining the details of the study in local or understandable language.

**4.10. Data collection and data confidentiality:** Data will be collected using a semi-structured questionnaire. Data will be kept anonymous, and confidentiality will be maintained.

**4.11. Plan of analysis:** Data will be collected through the Epicollect5 application. Cleaning will be done in excel and analyzed using STATA version 13. Categorical variables will be presented as proportion, and continuous variables will be presented as mean and standard deviation. Appropriate statistical tests of significance will be used. Matched pair analysis will be done using the McNemar chi-square test. A matched pair odds ratio will be estimated for ivermectin, HCQ and vitamin-C prophylaxis. The potential confounders which could not be matched will be adjusted during analysis with conditional logistic regression models. A secondary analysis will be carried out with different dosing's of ivermectin for its role in prevention.

**References:**

1. Center for System Science and Engineering, Johns Hopkins University. Coronavirus Covid-19 Global Cases. Available at https://www.arcgis.com/apps/opsdashboard/index.html#/bda7594740fd40299423467b48e9ecf6 accessed on 10 September 2020
2. Medscape.com. In Memoriam: Healthcare Workers Who Have Died of COVID-19. Available at <https://www.medscape.com/viewarticle/927976>
3. Wagstaf KM, Sivakumaran H, Heaton SM, Harrich D, Jans DA. Ivermectin is a specifc inhibitor of importin α/β-mediated nuclear importable to inhibit the replication of HIV-1 and dengue virus. Biochem J.2012;443(3):851–6. https://doi.org/10.1042/BJ20120150.
4. Mastrangelo E, Pezzullo M, De Burghgraeve T, Kaptein S, Pastorino B, Dallmeier K, de Lamballerie X, Neyts J, Hanson AM, Frick DN, Bolognesi M, Milani M. Ivermectin is a potent inhibitor of favivirus replication specifically targeting NS3 helicase activity: new prospects for an old drug. J Antimicrob Chemother. 2012;67(8):1884–94. https://doi.org/10.1093/jac/ dks147.
5. Lv C, Liu W, Wang B, Dang R, Qiu L, Ren J, Yan C, Yang Z, Wang X. Ivermectin inhibits DNA polymerase UL42 of pseudorabies virus entrance into the nucleus and proliferation of the virus in vitro and vivo. Antiviral Res. 2018;159:55–62. https://doi.org/10.1016/j.antiviral.2018.09.010.
6. Wang X, Lv C, Ji X, Wang B, Qiu L, Yang Z. Ivermectin treatment inhibits the replication of Porcine circovirus 2 (PCV2) in vitro and mitigates the impact of viral infection in piglets. Virus Res. 2019;2(263):80–6. https://doi. org/10.1016/j.virusres.2019.01.010.
7. Raza S, Shahin F, Zhai W, Li H, Alvisi G, Yang K, Chen X, Chen Y, Chen J, Hu C, Chen H, Guo A. Ivermectin inhibits bovine herpesvirus 1 DNA polymerase nuclear import and interferes with viral replication. Microorganisms.2020;8(3):E409. https://doi.org/10.3390/microorganisms8030409
8. Caly L, Druce JD, Catton MG, Jans DA, Wagstaf KM. The FDA-approved drug ivermectin inhibits the replication of SARS-CoV-2 in vitro. Antiviral Res. 2020. <https://doi.org/10.1016/j.antiviral.2020.104787>
9. Pacque M, Munoz B, Poetschke G, Foose J, Greene BM, Taylor HR: Pregnancyoutcome after inadvertent ivermectin treatment during community-based distribution.*Lancet* 1990,336(8729):1486-1489.
10. Anosike JC, Dozie IN, Ameh GI, Ukaga CN, Nwoke BE, Nzechukwu CT, UdujihOS, Nwosu DC. The varied beneficial effects of ivermectin (Mectizan) treatment, as observed within onchocerciasis foci in south-eastern Nigeria. Ann Trop MedParasitol. 2007 Oct;101(7):593-600.
11. NCT: 04422561. Use of Ivermectin as a Prophylactic Option in Asymptomatic Family Close Contacts with Patients of COVID-19.
12. nMaster 2.0 - Sample Size Software (http://www.cmc-biostatistics.ac.in/nmaster/).
