# Supplementary material for: Role of ivermectin in the prevention of SARS-CoV-2 infection among healthcare workers in India: A matched case-control study
Source: PLoS One. 2021 Feb 16;16(2):e0247163. doi: 10.1371/journal.pone.0247163 (PMC7886121; doi:10.1371/journal.pone.0247163)
Supplement: Attachment — Submitted filename: Response_Editorial_Query.docx [file pone.0247163.s003.docx]

To

The Editor PLOS ONE

Thanks for sharing the comments raised by the editorial staff. We want to place the following clarification regarding the study design.

The submitted manuscript is an outcome/observation from an ongoing health system intervention. We at AIIMS, Bhubaneswar, had a problem in hand of provisioning healthcare to patients on account of a large number of COVID-19 infections among healthcare staff. Ivermectin chemoprophylaxis (voluntarily) was advised based on the available evidence and recommendation of the institute task force on 11^th^ September 2020. Ivermectin chemoprophylaxis uptake among healthcare workers was encouraging.

An observational (matched case-control) study was planned to document the effect of recommended ivermectin chemoprophylaxis in October 2020. After ethical approval, the study was conducted in the 3^rd^ and 4^th^ week of October 2020. After selecting cases and controls, telephonic contact was established with each participant, and the history of ivermectin chemoprophylaxis was inquired.

The present manuscript describes the association of COVID19 infection with ivermectin chemoprophylaxis. Hence, it is a hypothesis-generating study that needs to be further confirmed by interventional studies as suggested. The protocol of the proposal and ethical approval letter is attached for your ready reference.

Thanking you

Dr Gitanjali Batmanabane, M.D. Ph.D.

Director, All India Institute of Medical Sciences

Bhubaneswar, Odisha, India – 751019

Email - director@aiimsbhubaneswar.edu.in
